# Supplementary material for: Development and Modification of Pre-miRNAs with a FRET Dye Pair for the Intracellular Visualization of Processing Intermediates That Are Generated in Cells
Source: Sensors (Basel). 2021 Mar 4;21(5):1785. doi: 10.3390/s21051785 (PMC7961592; doi:10.3390/s21051785)

Supplementary information

Development and modification of pre-miRNAs with a FRET dye pair for the intracellular visualization of processing intermediates that are generated in cells

Yukiko Kamiya*, Hiroshi Kamimoto, Hongyu Zhu, Hiroyuki Asanuma*

Department of Biomolecular Engineering, Graduate School of Engineering, Nagoya University, Furo-cho, Chikusa-ku, Nagoya 464-8603, Japan.

*Correspondence: yukikok@chembio.nagoya-u.ac.jp (Y.K.); asanuma@chembio.nagoya-u.ac.jp (H.A.); Tel: +81-52-789-2488

Table of Contents

1. Synthesis of phosphoramidite monomer of BO conjugated D-threoninol................S2
2. Supplementary figures..S4
3. Supplementary table..S10
4. References..S11
5. ^{1}H, ^{13}C, and ^{31}P NMR spectra of compounds...S12

Synthesis of phosphoramidite monomer of BO conjugated D-threoninol

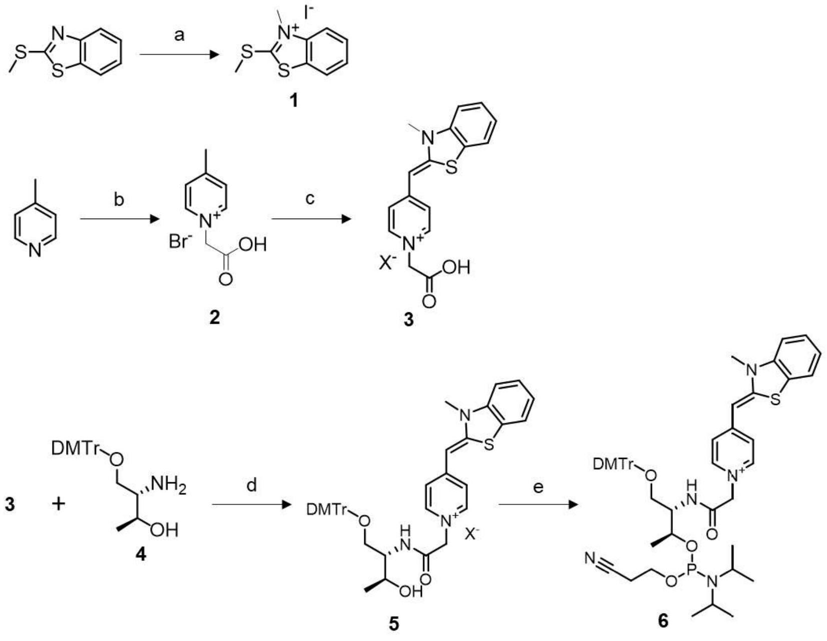

Scheme S1. Synthesis of phosphoramidite monomer of BO-conjugated D-threoninol

Reagents and conditions: a)CH_3I, EtOH, reflux, b) bromoacetic acid, EtOAc, c) Compound 1, Et_3N, CH_2Cl_2, reflux, under N_2, 60°C →r.t.; d) Compound 3 and 4, DMT-MM, DMF, r.t., overnight, 83%; e) 2-cyanoethyl *N*, *N*-diisopropylchlorophosphoramidite, Et_3N, CH_2Cl_2, 0°→r.t., 1.5h, 31%

Synthesis of compound 3: Compound **1** was synthesized according to the literature.[1] Ethyl acetate solution of bromoacetic acid (5.6 g, 40 mmol) was added to 4-methylpyridin (3.9 mL, 40 mmol). After overnight of stirring, 5.1 g of the crude product (compound **2, yield 55%**) was obtained as precipitate. The compounds **1** (2.9 g, 10 mmol) and **2** (2.3, 10 mmol) were suspended in dry CH_2Cl_2. The solution was refluxing under nitrogen at 60°C for 5 min, then triethylamine (7.0 mL) was gradually added. After the color of the reaction mixture turned orange, the mixture was stirred at r.t. for 2 h. The residue was subjected to silica gel column chromatography ($CHCl_3$/MeOH, 10:1 -> 3:1) to afford 0.65 g compound **3**.

^{1}H NMR [d_6-DMSO, 500MHz] δ=3.75 (s, 3H, N*CH_3*), 4.70 (s, 2H, C*H_2*COOH), 6.30 (S, 1H, (pyridine)-C*H*=(benzothiazole)), 7.30-8.75 (8H, pyridine-benzothiazole)

^{13}C NMR [d_6-DMSO, 125MHz] δ=170.38, 157.85, 151.25, 141.72, 140.63, 127.58, 123.82, 123.40, 121.96, 111.28, 89.26, 60.55, 31.72

HRMS (FAB): Calcd for compound 3 (M^+) 299.0849. Found 299.0575

Synthesis of compound 5: Compound 3 (1.4 g, 3.6 mmol) was dissolved in DMF and then coupled with compound 4 (1.5 g, 3.6 mmol) in the presence of DMT-MM (5.0 mmol). After the reaction, the mixture was diluted with $CHCl_3$ and the organic layer was separated with saturated $NaHCO_3$ and with H_2O. The mixture was dried over $MgSO_4$. The filtrated solvent was dried *in vacuo.* The clude mixture was subjected to silica gel column chromatography ($CHCl_3$ 100% -> $CHCl_3$/MeOH, 15:1) to afford 1.9 g of compound 5 (yield 64%).

^{1}H NMR [d_6-DMSO, 500MHz] δ=1.05 (d, 3H, C-CH3), 2.90-3.15 (m, 2H, DMTO-CH2-), 3.75 (s, 6H, -OCH3), 3.80 (s, 3H, -NCH3), 3.85-4.05 (m, 1H, HO-CH-CH3), 4.90-5.10 (m, 2H, -CH2-COOH), 6.30 (s, 1H, (pyridine)-CH=(benzothiazole)), 6.80-8.25 (21H, benzene)

^{13}C-NMR [d_6-DMSO, 125 MHz] δ=164.90, 157.41, 156.99, 149.98, 144.37, 141.73, 139.90, 135.15, 129.67, 127.20, 127.12, 126.00, 123.07, 122.81, 121.99, 112.55, 111.60, 89.27, 84.65, 78.60, 64.06, 62.17, 58.28, 54.43, 54.07, 32.34, 19.78,

HRMS (FAB): Calcd for compound 5 (M^+) 688.2840. Found 688.2824

Synthesis of compound 6: Compound 5 (0.82 g, 1.0 mmol) was dissolved in dry CH_2Cl_2 (5 mL) and Et_3N (0.69 mL) and cooled on ice under nitrogen. Then 0.45 mL (2.0 mmol) of 2-cyanoethyl *N,N*-diisopropylchlorophosphoramidite was added dropwise. The crude mixture was subjected to silica gel column chromatography ($CHCl_3$/MeOH, 20 : 1 including 3% Et_3N) to afford 0.42 g of compound 8 (yield 42%).

^{31}P-NMR [$CDCl_3$, 202 MHz] δ= 147.62, 147.19

HRMS (FAB): Calcd for compound 7 (M^+) 888.3918. Found 888.3476

Supplementary table and figures

(a)

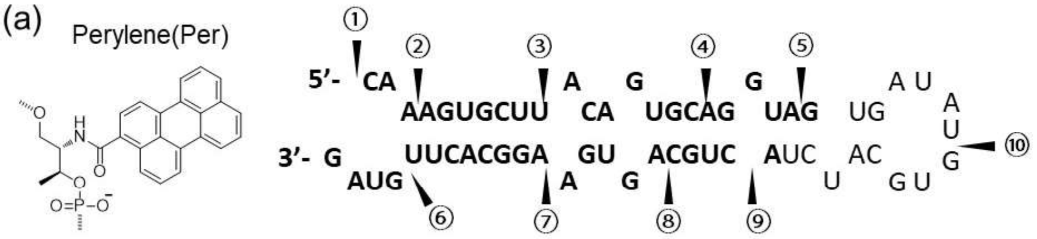

(b)

	Sequences	T_m (°C)	ΔT_m (°C)
①	5' – **Per**-CAAAGUGCUUACAGUGCAGGUAGUG – 3'<br>3' – GAUGUUCACGGAAGUGACGUCAUCUACGUGUAUA **p**– 5'	64.0	2.6
②	5' –CAA-**Per**-AGUGCUUACAGUGCAGGUAGUG – 3'<br>3' – GAUGUUCACGGAAGUGACGUCAUCUACGUGUAUA **p**– 5'	65.3	4.0
③	5' –CAAAGUGCU-**Per**-UACAGUGCAGGUAGUG – 3'<br>3' – GAUGUUCACGGAAGUGACGUCAUCUACGUGUAUA **p**– 5'	66.4	5.0
④	5' –CAAAGUGCUUACAGUGC-**Per**-AGGUAGUG – 3'<br>3' – GAUGUUCACGGAAGUGACGUCAUCUACGUGUAUA **p**– 5'	64.4	3.0
⑤	5' –CAAAGUGCUUACAGUGCAGGUA-**Per**-GUG – 3'<br>3' – GAUGUUCACGGAAGUGACGUCAUCUACGUGUAUA **p**– 5'	64.9	3.6
⑥	5' – CAAAGUGCUUACAGUGCAGGUAGUG – 3'<br>3' – GAUG-**Per**-UUCACGGAAGUGACGUCAUCUACGUGUAUA **p**– 5'	64.9	3.5
⑦	5' – CAAAGUGCUUACAGUGCAGGUAGUG – 3'<br>3' – GAUGUUCACGG-**Per**-AAGUGACGUCAUCUACGUGUAUA **p**– 5'	64.8	3.4
⑧	5' – CAAAGUGCUUACAGUGCAGGUAGUG – 3'<br>3' – GAUGUUCACGGAAGUGA-**Per**-CGUCAUCUACGUGUAUA **p**– 5'	62.9	1.5
⑨	5' – CAAAGUGCUUACAGUGCAGGUAGUG – 3'<br>3' – GAUGUUCACGGAAGUGACGUC-**Per**-AUCUACGUGUAUA **p**– 5'	66.7	5.4
⑩	5' – CAAAGUGCUUACAGUGCAGGUAGUG – 3'<br>3' – GAUGUUCACGGAAGUGACGUCAUCUACGUG-**Per**-UAUA **p**– 5'	62.9	0.9

(c)

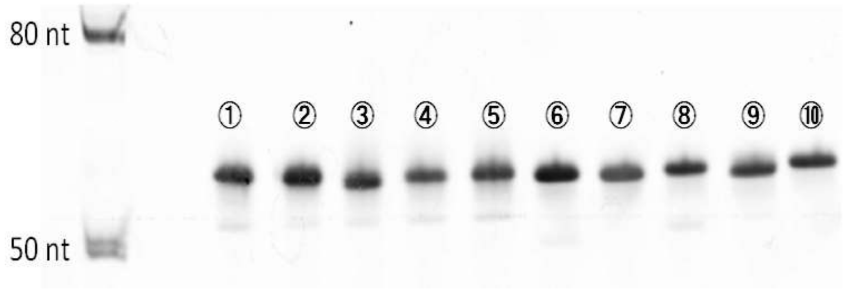

Figure S1 Preparation of Per-modified pre-miR17: (a) Chemical structure of Per that was inserted into pre-miR17. The modification positions are shown as indications. (b)Sequence of RNAs and T_ms, and (c) ligation products analyzed by PAGE are indicated. **p** in the sequences indicates phosphate group. Measurement condition of T_m was follows: 10 mM phosphate buffer (pH 7.0), 100 mM NaCl, 1.0 μM pre-miR17, 260 nm, 0.5°C/min. ΔT_m was the difference between T_ms of native pre-miR17 and the Per-modified pre-miR17. The purified Per-modified pre-miR17s were analyzed by PAGE and detected by FLA 9500 with 473 nm excitation and LPB filter.

(a)

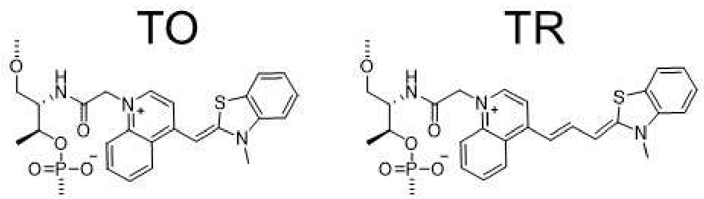

(b)

A5TO	3'- UUGG**TO**AAUGGGUGGAGUACAUAGA -5'
S5TR	5'- AACC**TR**UUACCCACCUCAUGUAUCU -3'
S7TR	5'- AACCUU**TR**ACCCACCUCAUGUAUCU -3'
S9TR	5'- AACCUUAC**TR**CCACCUCAUGUAUCU -3'
S11TR	5'- AACCUUACCC**TR**ACCUCAUGUAUCU -3'

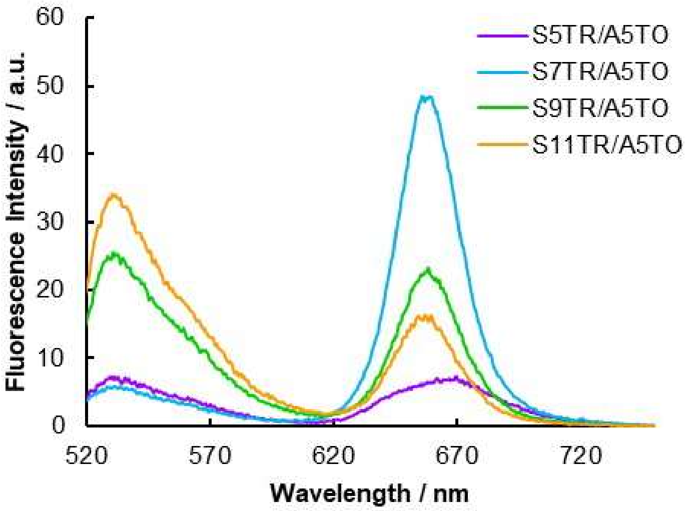

Figure S2 Fluorescent emission spectra of dsRNA possessing the TO and TR at different position. (a) Chemical structures of TO and TR that were inserted into dsRNA through D-threoninol and the (b) sequence of RNAs. Fluorescent emission spectra of dsRNA at 20°C. The solutions conditions are as follows: a 10 mM phosphate buffer, 100 mM NaCl, pH 7.0, 1.0 μM A5TO, 1.2 μM RNA possessing TR. The fluorescence spectra of dsRNAs were recorded upon excitation at 510 nm in the gain setting as high. Fluorescence intensity is given in arbitrary units (a.u.). Phosphoramidite D-threoninol monomers of TR was synthesized as described with modifications in the literature.[2] The MS data for ODNs were as follows: m/z: S5TR: Obsd. 7669.30, Calcd. 7669.07, S7TR: Obsd. 7669.10, Calcd. 7669.07, S9TR: Obsd. 7673.54, Calcd. 7669.07, S11TR: Obsd. 7672.59, Calcd. 7669.07

(a)

	S7Per/A5TO	S7Per/A5Cy3	S7BO/A5TO	S7BO/A5Cy3
T_m (°C)	79.0	78.1	75.5	74.8

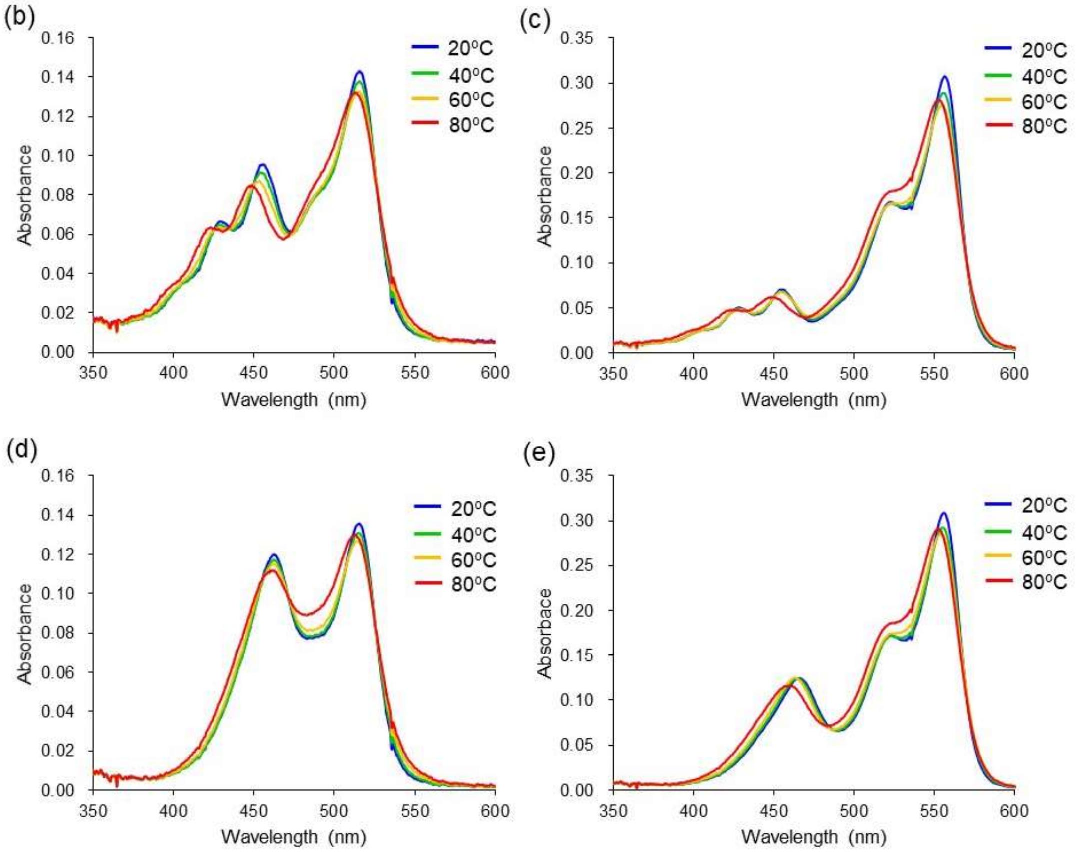

Figure S3 T_ms (a) and UV-Vis spectra of S7Per/A5TO (b), S7Per/A5Cy3 (c), S7BO/A5TO (d), and S7BO/A5Cy3 (e). The solution conditions are as follows: a 10 mM phosphate buffer, 100 mM NaCl, pH 7.0, 2.0 μM RNA possessing a donor fluorophore, 2.4 μM RNA possessing an acceptor fluorophore. The T_m of unmodified dsRNA was 78.3°C. The λmax of dyes in the FRET-dsRNA were red shifted when the dsRNAs were formed, indicating that the dyes were intercalated within dsRNA.

(a)

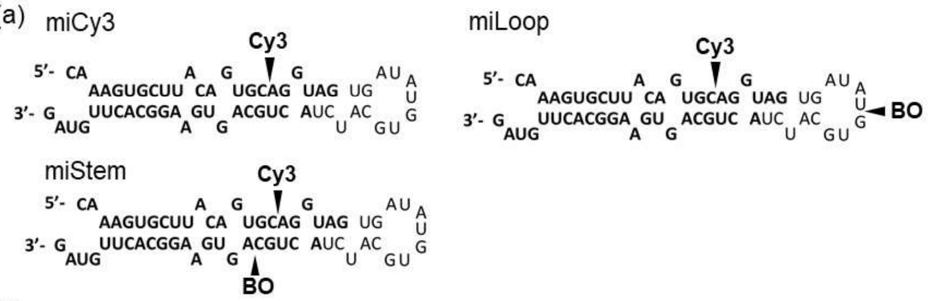

(b)

	Sequences
miCy3	5' – CAAAGUGCUUACAGUGC-**Cy3**-AGGUAGUG – 3'
	3' – GAUGUUCACGGAAGUGACGUCAUCUACGUGUAUA **p**– 5'
miLoop	5' – CAAAGUGCUUACAGUGC-**Cy3**-AGGUAGUG – 3'
	3' – GAUGUUCACGGAAGUGACGUCAUCUACGUG-**BO**-UAUA p– 5'
miStem	5' –CAAAGUGCUUACAGUGC-**Cy3**-AGGUAGUG – 3'
	3' – GAUGUUCACGGAAGUGA-**BO**-CGUCAUCUACGUGUAUA p– 5'

(c)

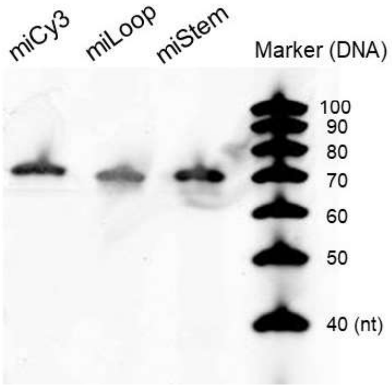

(d)

	T_m (°C)	ΔT_m (°C)
miLoop	61.4	-0.5
miStem	58.7	-2.7

Figure S4 Preparation of Cy3 or BO-Cy3 modified pre-miR17: (a) Design of the modified pre-miR17, (b) sequences of RNAs, (c) ligation products, and (d) T_ms of miCy3, miLoop, and miStem. The purified pre-miR17s were analyzed by PAGE and detected by FLA 9500 with 473 nm excitation and LPB filter. Measurement condition of T_m was follows: 10 mM phosphate buffer (pH 7.0), 100 mM NaCl, 1.0 µM pre-miR17, 260 nm, 0.5 °C/min. ΔT_m was the difference between T_ms of native pre-miR17 and the FRET-modified pre-miR17.

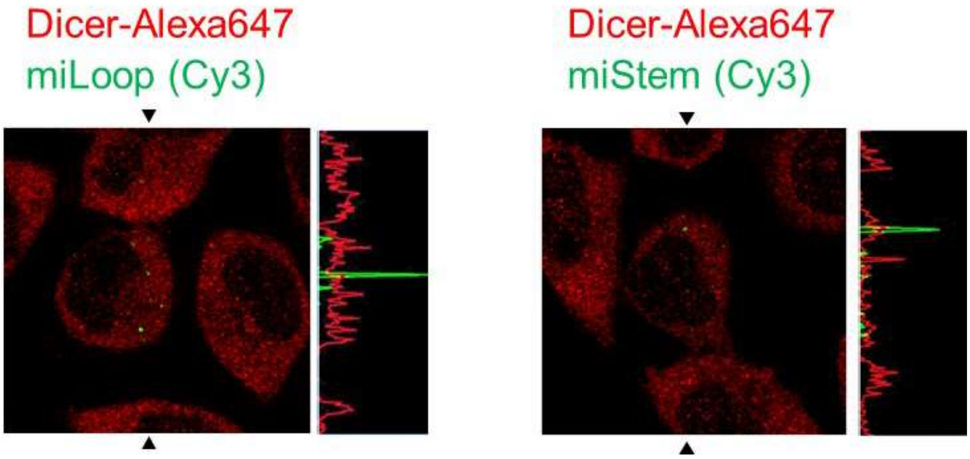

Figure S5 Immunostaining of Dicer in the HeLa cells transfected with the miLoop or miStem. The fluorescence signals of those that were transfected by the beads loading method were observed by confocal laser microscopy. Endogenous Dicer was visualized by an anti-Dicer and Alexa648-conjugated secondary antibodies. Pictures of Cy3 (green) and Alexa 647 (red) signals were merged. The signal strength of the section between the black allows in the pictures are displayed on the right side of each image. The fluorescent signals were recorded as follows. Cy3: Ex = 543 nm, Em = 555-625 nm; Alexa647, Ex = 633 nm, Em = 645-745 nm.

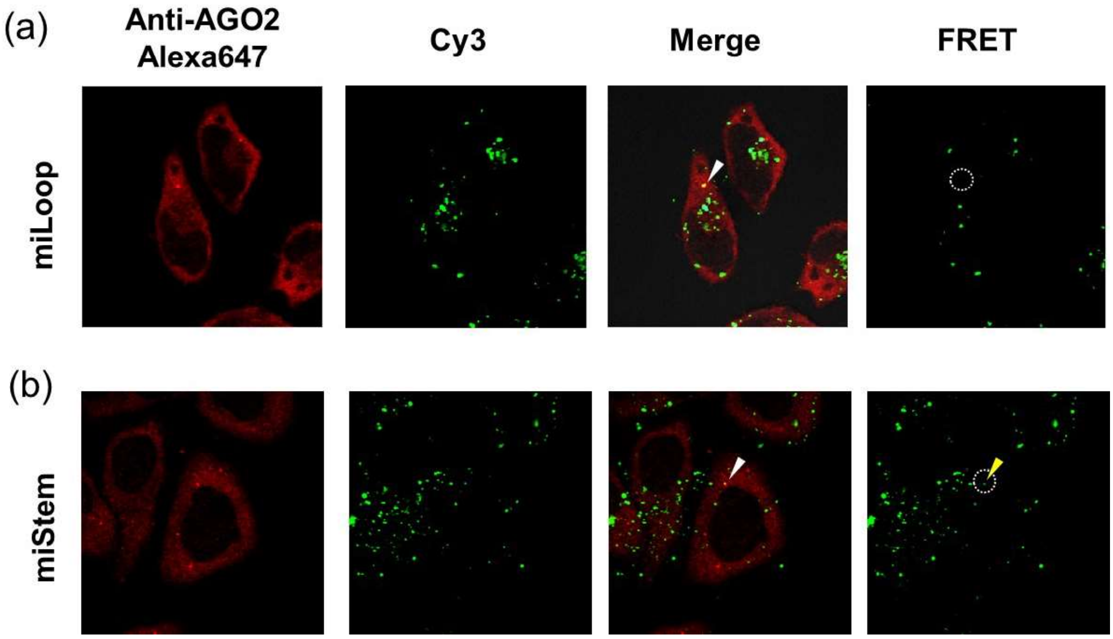

Figure S6 Confocal laser fluorescent microscopy images of HeLa cells with lipofection of miLoop (a) or miStem (b). The FRET modified pre-miRNAs (30 nM) were transfected by using Lipofectamin® 3000 to the HeLa cells. Endogenous AGO2 was visualized by an anti-AGO2-antibody and Alexa648-conjugated secondary antibodies. Cy3 and Alexa 647 signals are shown in green and red, respectively. In the images, many complexes of lipid particle with the pre-miR17 were observed. The overlays of the red and green images show the co-localization of Cy3 with AGO2, as indicated by white arrows. The foci that were detected by FRET from miStem is represented by the yellow arrow. Dotted circle indicated in FRET panel is the place where co-localization signal between Cy3 and Alexa 647 was observed in the merge panel. Fluorescent signals were recorded as follows. Cy3: Ex = 543 nm, Em = 555-625 nm; Alexa647, Ex = 633 nm, Em = 645-745; FRET : Ex = 458 nm, Em = 555-625 nm.

Table S1 MS data of synthesized RNAs

	Obsd.(m/z)	Calcd. for [M+H+] (m/z)
S7Per	7587.65	7590.04
S7BO	7594.21	7593.04
A5TO	7989.24	7986.14
A5Cy3	8033.27	8038.26

References

1. Hara, Y.; Fujii, T.; Kashida, H.; Sekiguchi, K.; Liang, X.; Niwa, K.; Takase, T.; Yoshida, Y.; Asanuma, H. Coherent quenching of a fluorophore for the design of a highly sensitive in-stem molecular beacon. *Angew Chem Int Ed Engl* **2010**, *49*, 5502-5506.
2. Holzhauser, C.; Berndl, S.; Menacher, F.; Breunig, M.; Gopferich, A.; Wagenknecht, H.A. Synthesis and Optical Properties of Cyanine Dyes as Fluorescent DNA Base Substitutions for Live Cell Imaging. *Eur. J. Org. Chem.* **2010**, *2010*, 1239-1248.

Compound 3
^{1}H-NMR

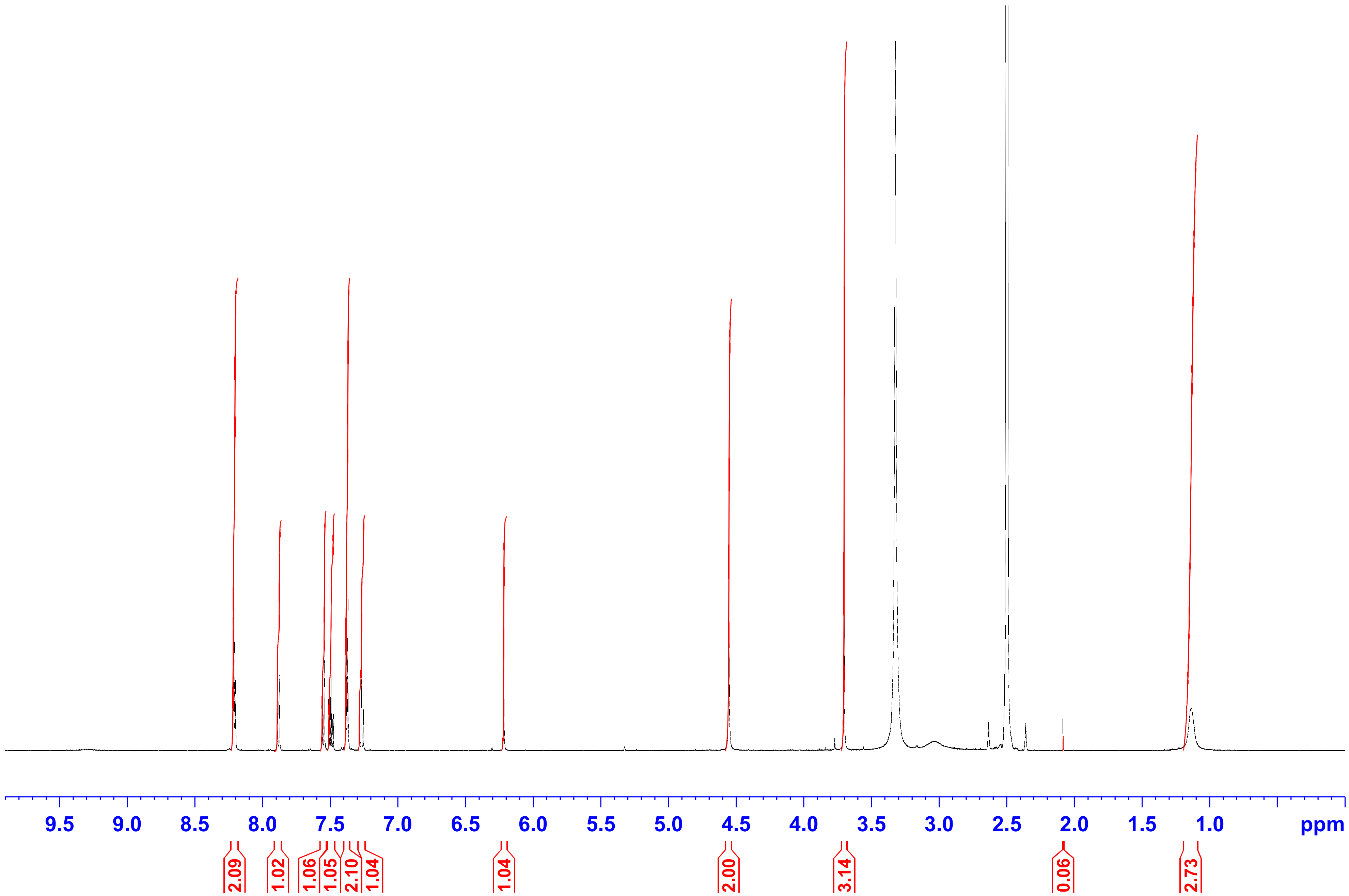

Compound 3

^{13}C-NMR

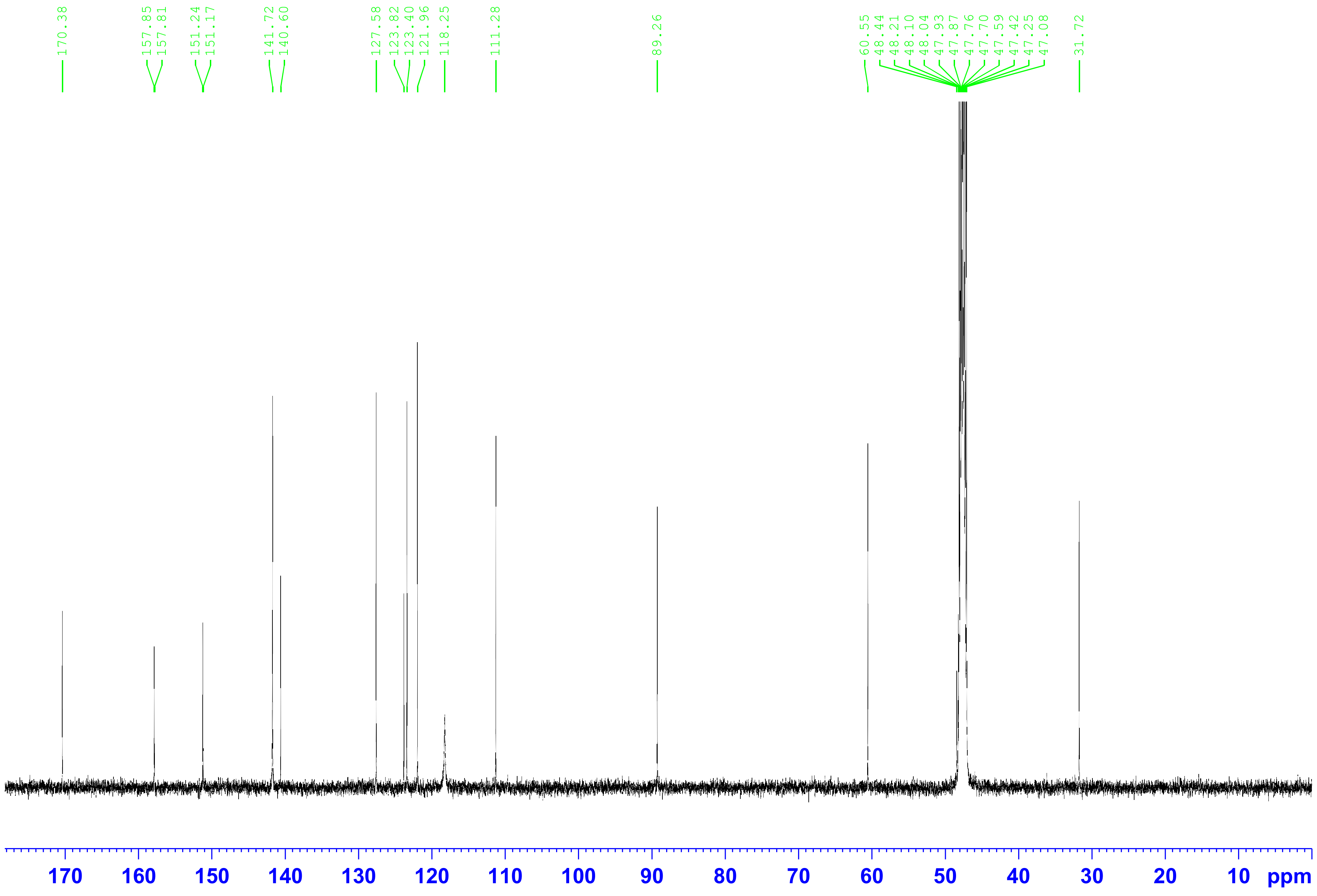

Compound 5
^{1}H-NMR

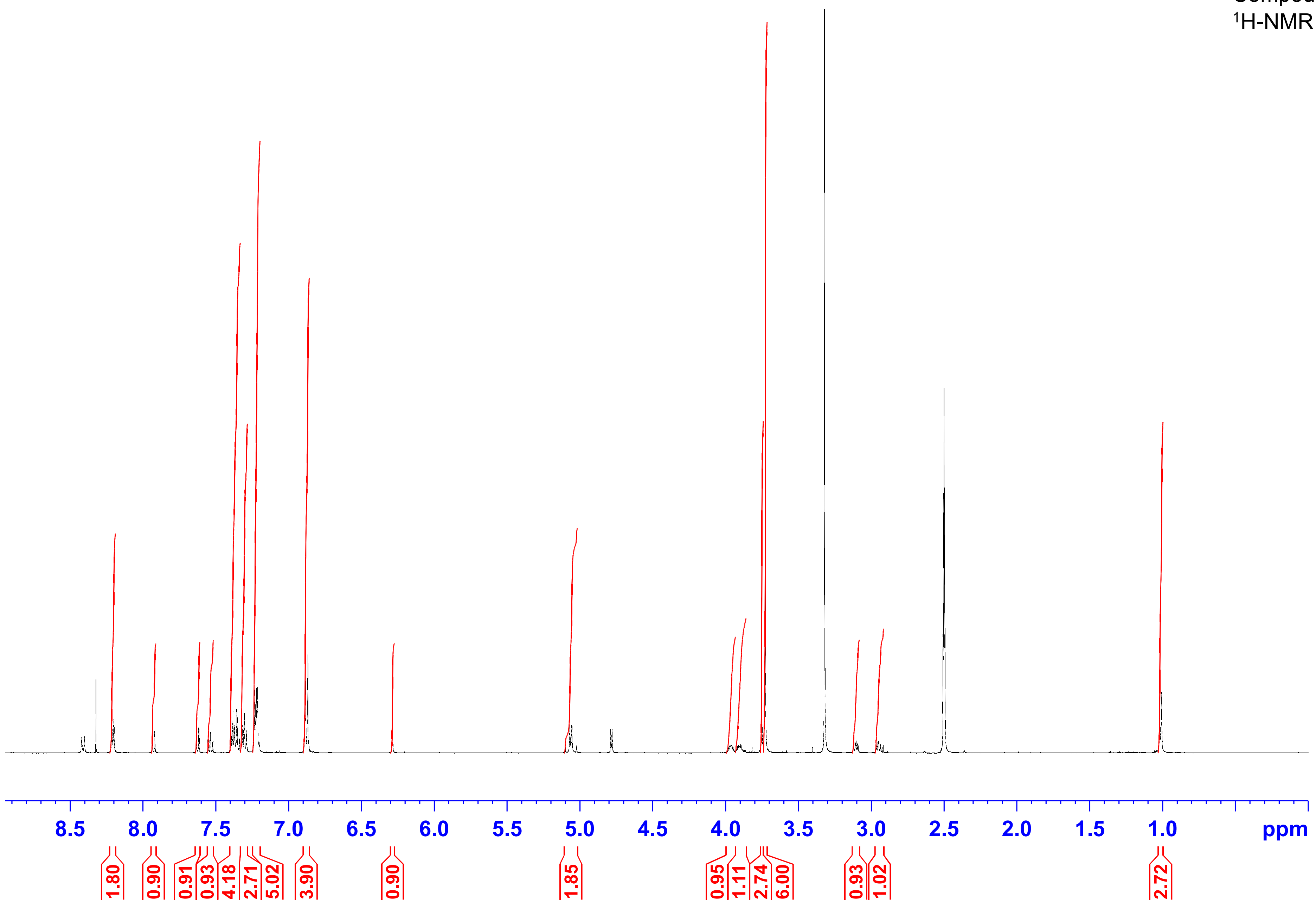

Compound 5

^{13}C-NMR

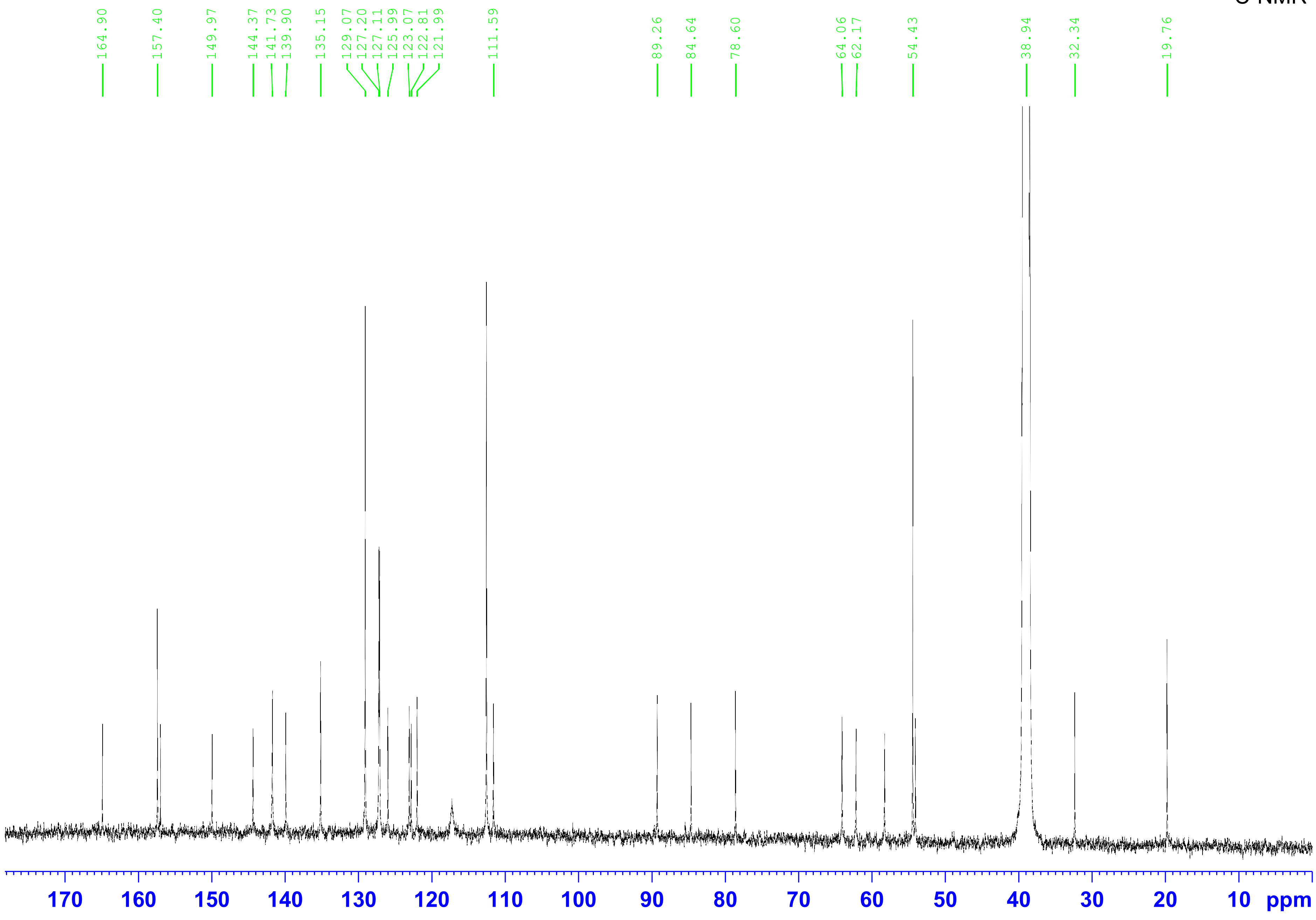

Compound 6
^{31}P-NMR

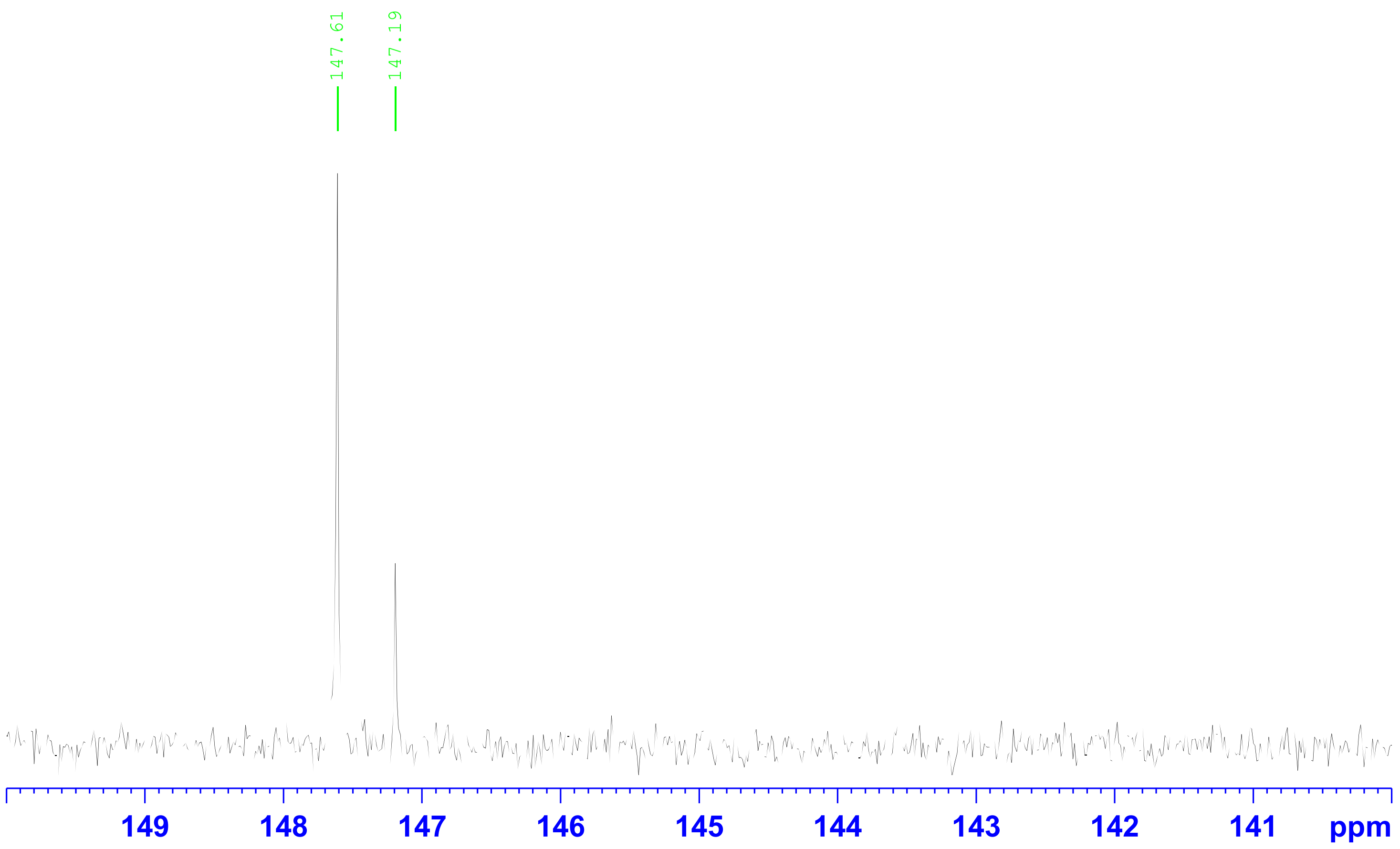

Supplement: Supplementary file 1 [file sensors-21-01785-s001.pdf]
